# Tumor-infiltrating CD45RO^+^ Memory T Lymphocytes Predict Favorable Clinical Outcome in Solid Tumors

**DOI:** 10.1038/s41598-017-11122-2

**Published:** 2017-09-04

**Authors:** Guoming Hu, Shimin Wang

**Affiliations:** 1 Department of General Surgery (Breast and Thyroid Surgery), Shaoxing People’s Hospital; Shaoxing Hospital of Zhejiang University, 312000 Zhejiang, China; 2 Department of Nephrology, Shaoxing People’s Hospital, Shaoxing Hospital of Zhejiang University, 312000 Zhejiang, China

## Abstract

The prognostic role of tumor-infiltrating CD45RO^+^ memory T lymphocytes (CD45RO^+^ T cells) in human solid tumors remains controversial. Herein, we conducted a meta-analysis including 25 published studies with 4720 patients identified from PubMed and EBSCO to assess the prognostic impact of tumor-infiltrating CD45RO^+^ T cells in human solid tumors. We found that CD45RO^+^ T cell infiltration was significantly associated with improved overall survival (OS) and disease-free survival (DFS) in all types of solid tumors. In stratified analyses, CD45RO^+^ T cell infiltration significantly improved 1-year, 3-year and 5-year OS in colorectal, gastric and esophageal cancer, but only 5-year OS in hepatocellular carcinoma. And these cells were positively associated with 1-year, 3-year and 5-year DFS in hepatocellular, colorectal and esophageal cancer. In addition, high density of intratumoral CD45RO^+^ T cells inversely correlated with TNM stage of solid tumor. In conclusion, CD45RO^+^ memory T lymphocyte infiltration leads to a favorable clinical outcome in solid tumors, implicating that it is a valuable biomarker for prognostic prediction for human solid malignances.

## Introduction

Accumulating evidence has demonstrated that tumor microenvironment (TME) linked closely with the initiation, promotion, and progression of cancer^1^. Tumor-infiltrating lymphocytes (TILs) are the major component of TME^2^. Previous studies have revealed that TILs were significantly positively associated with survival of solid tumors^3^. However, it is important to distinguish among different types of T lymphocytes as they may play differential roles in the TME. CD45RO^+^ memory T lymphocytes (CD45RO^+^ T cells), as the important component of TILs, have been demonstrated to play specific and significant roles in a number of human cancers.

CD45 is known as the leukocyte common antigen, and functions as a tyrosine phosphatase in leukocyte signaling. The expression of different CD45 isoforms is cell - type specific and depends on the state of activation and the stage of differentiation of cells. CD45RO is the most suitable single marker for human memory T cells, that can finely represent the activation status of T cells^4^. CD45RO^+^ T cells often increased in solid tumors. Recent studies have associated CD45RO^+^ T cells and cancer prognosis, but their results were controversial. Thus, an in-depth assessment is warranted. Moreover, the potential of these cells as an effective biomarker in prognostic prediction is necessary to be explored.

Here, we performed this meta-analysis to test overall survival (OS) and disease-free survival (DFS) as outcomes in patients with solid tumor with known intratumoral CD45RO^+^ T cell density evaluated by immunohistochemistry (IHC). The aim of this study was to quantitatively summarize the association between CD45RO^+^ T cell infiltration and clinical outcomes in cancer patients, and thereby provided more evidence on the clinical value of tumor-infiltrating CD45RO^ + ^T cells as a prognostic biomarker for solid malignances.

## Materials and Methods

### Search strategy

We searched PubMed and EBSCO for studies assessing the density of CD45RO^+^ T cells in tumor tissue and survival in patients with solid tumor from 1996 to January 15th 2017. The searching keywords were (CD45RO [Title/Abstract]) AND (neoplasms [Title/Abstract] OR cancer [Title/Abstract] OR tumor [Title/Abstract] OR carcinoma [Title/Abstract]). A total of 724 and 1847 entries were identified in PubMed and EBSCO respectively.

### Inclusion and exclusion criteria

Inclusion criteria of the meta-analysis were: studies included must have (1) been published as original articles; (2) evaluated human subjects; (3) CD45RO^+^ T cells in tumor specimens were evaluated by IHC; (4) provided Kaplan – Meier curves of high and low CD45RO^+^ T cell density with overall survival (OS), and/or disease-free survival (DFS), or relapse-free survival (RFS); (5) published in English.

We excluded studies that were not published as full texts, including commentary, case report, conference abstracts and letters to editors, studies that not report sufficient data to estimate survival rates; studies that evaluated CD45RO^+^ T cells with Flow Cytometry (FCM) or real-time reverse transcription polymerase chain reaction (RT-PCR), detected CD45RO^+^ T cells in metastases and not in tumor tissues.

### Endpoints

OS and DFS (or RFS) were the endpoints used in this meta-analysis. OS was recorded as the primary endpoint, and the second endpoint was DFS (or RFS). Cut-offs of CD45RO^+^ T cell density defined by individual studies classified cancer patients into high- and low- groups.

### Data extraction

Two authors (G.M.H. and S.M.W.) independently reviewed and extracted data using predefined data abstraction form from each eligible study. Extracted information included first author’s name, publication year, country, number of patients, median age, gender, Tumor, Lymph Node, Metastasis (TNM) stage, tumor differentiation, time of follow-up, technique used to quantify CD45RO^+^ T cells, and cut-off value to determine high CD45RO^+^ T cell density. OS, DFS (or RFS) and clinicopathological data were extracted from the text, tables, or Kaplan – Meier curves for both high and low CD45RO^+^ T cell density groups.

### Quality assessment

The studies included in the meta-analysis were cohort studies. The quality of each included study was assessed using Newcastle–Ottawa Scale (NOS) by two independent authors^5^. The studies with 6 scores or more were classified as high quality studies. A consensus NOS score for each item was achieved.

### Statistical Analysis

Extracted data were combined into a meta-analysis using STATA 12.0 analysis software (Stata Corporation, College Station, TX, USA). Statistical heterogeneity was assessed using the chi-squared based Q-test or the *I* ^2^ method^6^. Data were combined according to the random-effect model in the presence of heterogeneity^7^, otherwise, the fixed-effect model was performed^8^). Sensitivity analysis was employed to assess the influence of each study on the pooled result. Begg’s funnel plot and Egger’s test^9^ were calculated to investigate potential publication bias. All *P* values were two-sided and less than 0.05 are considered statistically significant.

## Result

### Search results and description of studies

**Figure 1:**
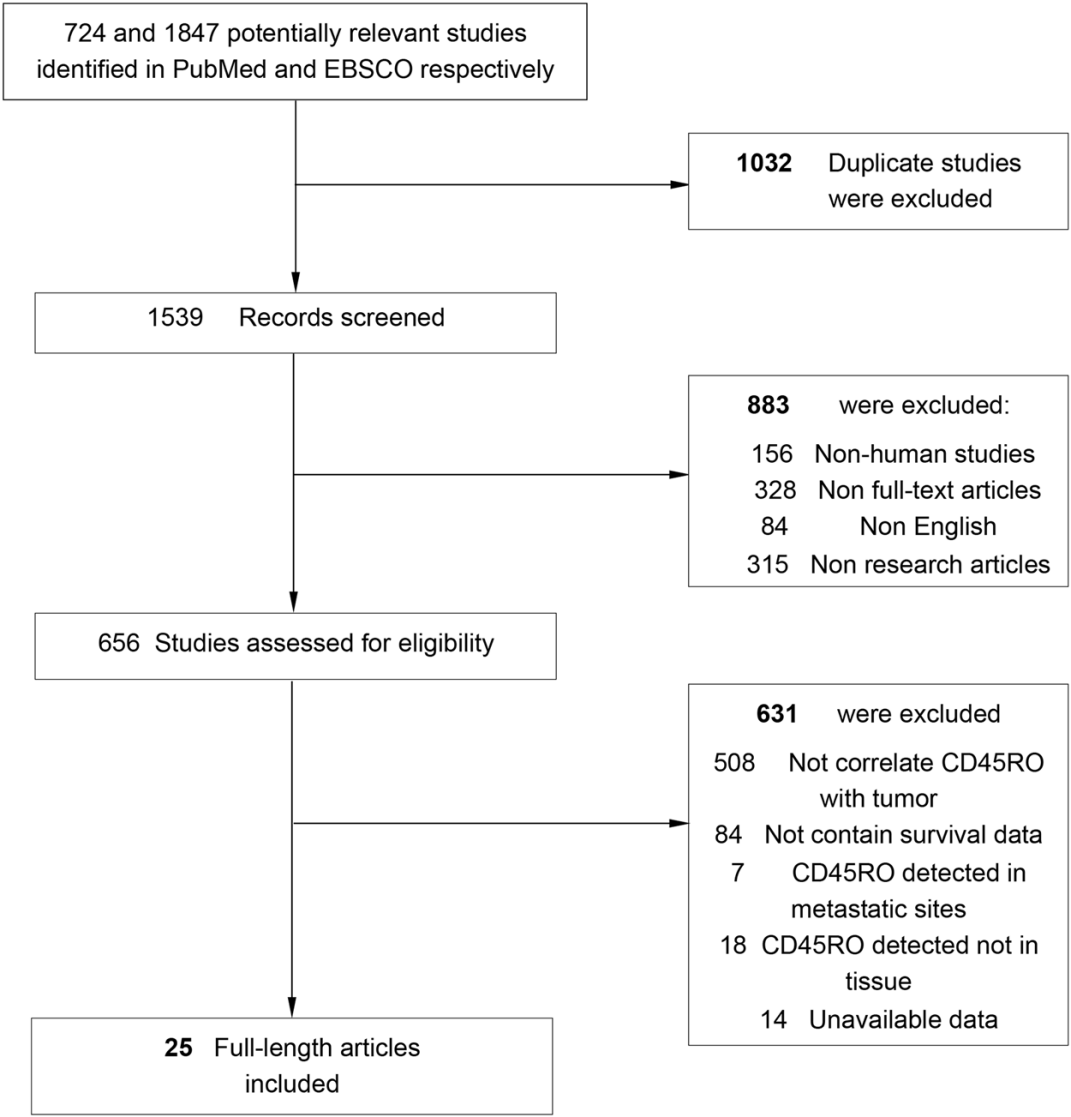
Flow chart diagram of study selection.

Literature searches yield 2571 records and the results were shown in Fig. 1. 25 studies containing 4720 patients with solid tumor were identified for the assessment of tumor-infiltrating CD45RO^+^ T cells^10–34^. All the studies were evaluated by the Newcastle–Ottawa Scale (NOS), and were in accordance with the inclusion criteria and suitable for data consolidation. Characteristics of included studies for OS, DFS and clinicopathological features such as TNM stage, tumor differentiation *et al*. were shown in Table 1 and Table S1 respectively.

**Table 1:** Main characteristics of the included studies.

Study	Year	Tumor type	No. of Patients	Male/Female	median age (range) (year)	CD45RO^+^ T: High/Low	Tumor stage	median follow-up date (months)	Survival	Quality Score (NOS)
Yajima, R. *et al*.^10^	2016	Breast cancer	98	0/98	NR	49/49	I–III	84	OS, DFS	6
Zhang, Z. *et al*.^11^	2015	Ovarian cancer	33	0/33	50.33 ± 10.84	31/2	I–IV	35 (1, 126)	OS	6
Paulsen, E. E. *et al*.^12^	2015	Non-small cell lung cancer	504	343/161	≤65: 45%; >65: 55%	423/81	I–III	61 (0.3, 81.6)	OS	7
Gao, Q. *et al*.^13^	2012	Hepatocellular carcinoma	206	186/20	≤49: 50.5%; >49: 49.5%	103/103	I–III	48.1 (3.4, 111.9)	OS, DFS	8
Hotta, K. *et al*.^14^	2011	Renal cell carcinoma	105	76/29	<60: 34.3%; ≥60: 65.7%	52/53	I–IV	15.9 (2, 52.5)	OS	7
Wakatsuki, K. *et al*.^15^	2013	Gastric cancer	74	54/20	65 (36, 84)	37/37	I–IV	NR	OS, DFS	7
Lee, H. E. *et al*.^16^	2008	Gastric cancer	220	156/64	<66: 80.5%; ≥66: 19.5%	65/155	I–IV	NR	OS	7
Li, Y. W. *et al*.^17^	2009	Hepatocellular carcinoma	302	260/42	≤60: 76.8%; >60: 23.2%	150/152	I–III	58 (2, 121)	OS, DFS	7
Enomoto, K. *et al*.^18^	2012	Esophageal cancer	105	85/20	61·4 (42, 78)	54/51	I–IV	NR	OS, DFS	7
Chang, K. C. *et al*.^19^	2007	Diffuse Large B-Cell Lymphomas	48	27/21	≤60: 39.6%; >60: 60.4%	18 /30	I–IV	NR	OS	6
Anraku, M. *et al*.^20^	2008	Malignant pleural mesothelioma	32	28/4	59 (21, 74)	15/14	II–IV	35 (9, 63)	OS	6
Rauser, S. *et al*.^21^	2010	Esophageal cancer	110	102/8	63.6 (33, 83)	93/17	I–IV	33 (0.8, 164)	OS, DFS	7
Brunner, S. *et al*.^22^	2014	Colorectal cancer	121	NR	62 (53, 68)	63/58	IV	NR	OS	6
de Jong, R. A. *et al* ^23^	2009	Endometrial cancer	298	0/298	65 (32, 89)	181 /117	I–IV	4.4 (0, 21.5)	OS	6
Zhang, Y. *et al*.^24^	2016	Gallbladder carcinoma	98	51/47	63 (39, 88)	48/50	I–IV	NR	OS	7
Lee, W. S. *et al*.^25^	2010	Colorectal cancer	53	29/34	≤60: 47.2%; >60: 52.8%	25/28	II	NR	OS, DFS	7
Galon, J. *et al*.^26^	2006	Colorectal cancer	243	NR	NR	121/122	I–IV	45.3	OS, DFS	7
Peng, R. Q. *et al*.^27^	2010	Colorectal cancer	72	40/32	<60: 45.8%; ≥60: 54.2%	54/18	IIIB	NR	OS	6
Wang, L. *et al*.^28^	2015	Colorectal cancer	185	110/75	58 (22, 85)	91/94	I–III	NR	OS	7
Pages, F. *et al*.^29^	2005	Colorectal cancer	336	NR	NR	160/176	Dukes’ A–D	44.5	OS, DFS	8
Koelzer, V. H. *et al*.^30^	2014	Colorectal cancer	130	80/50	NR	65/65	I–IV	NR	OS	7
Kim, Y. H. *et al*.^31^	2015	Colorectal cancer	218	133/85	<65: 54.6%; ≥65: 45.4%	103/115	I–IV	NR	OS	7
Lee, W. S. *et al* ^32^	2013	Colorectal cancer	94	NR	NR	46/48	IV	39.1 (3, 75)	OS	7
Nosho, K. *et al*.^33^	2010	Colorectal cancer	738	NR	NR	356/382	I–IV	11.6	OS	7
Chen, Y. F. *et al*.^34^	2016	Colorectal cancer	300	158/142	<60: 48%; ≥60: 52%	112/188	I–IV	62.9 ± 29.3	OS, DFS	8

### Meta-analyses

#### Overall survival (OS)

**Figure 2:**
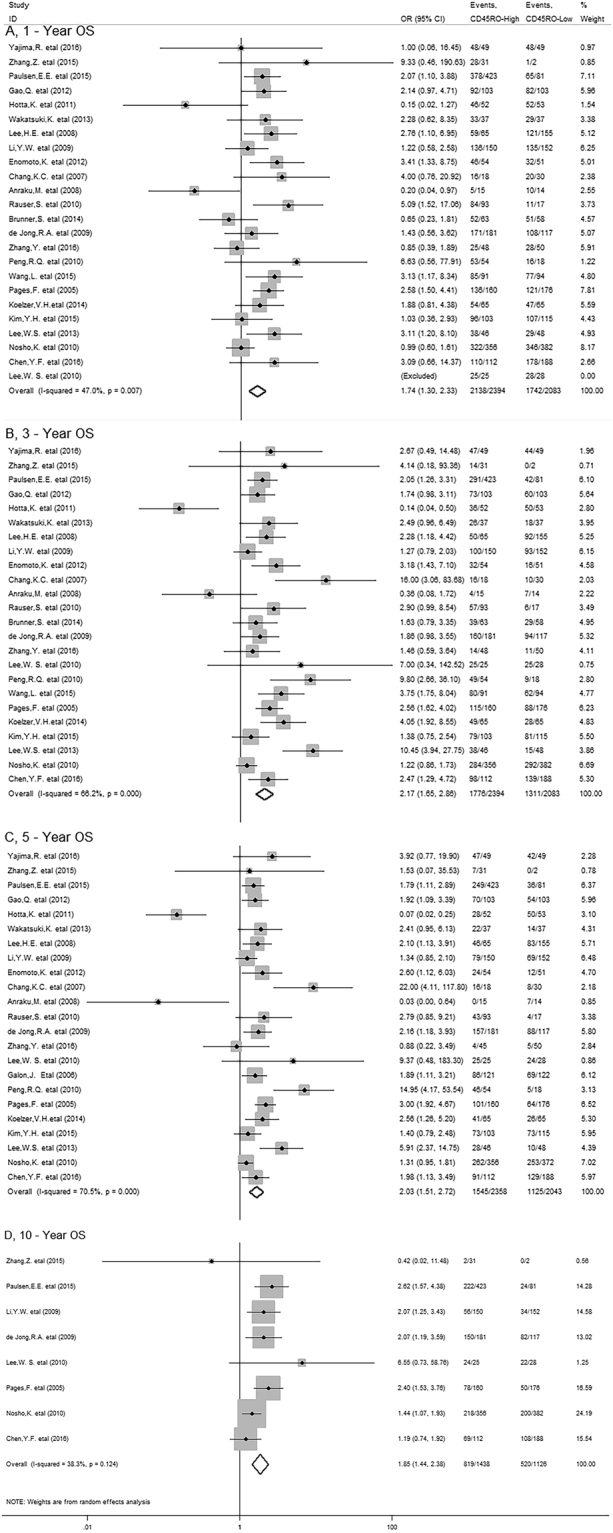
Forest plots describing ORs of the association between CD45RO^+^ T cell infiltration and OS at 1-year, 3-year, 5-year and 10-year.

The meta-analysis showed that CD45RO^+^ T cells infiltrating into tumor was significantly associated with better 1-year (OR = 1.74, 95% CI 1.30 to 2.33, *P* = 0.000) and 3-year OS (OR = 2.17, 95% CI 1.65 to 2.86, *P* = 0.000) in patients with solid tumor (Fig. 2A and B); Similar results were observed between CD45RO^+^ T cells and 5-year (OR = 2.03, 95% CI 1.51 to 2.72, *P* = 0.000) and 10-year OS (OR = 1.85, 95% CI 1.44 to 2.38, *P* = 0.000) (Fig. 2C and D).

**Figure 3:**
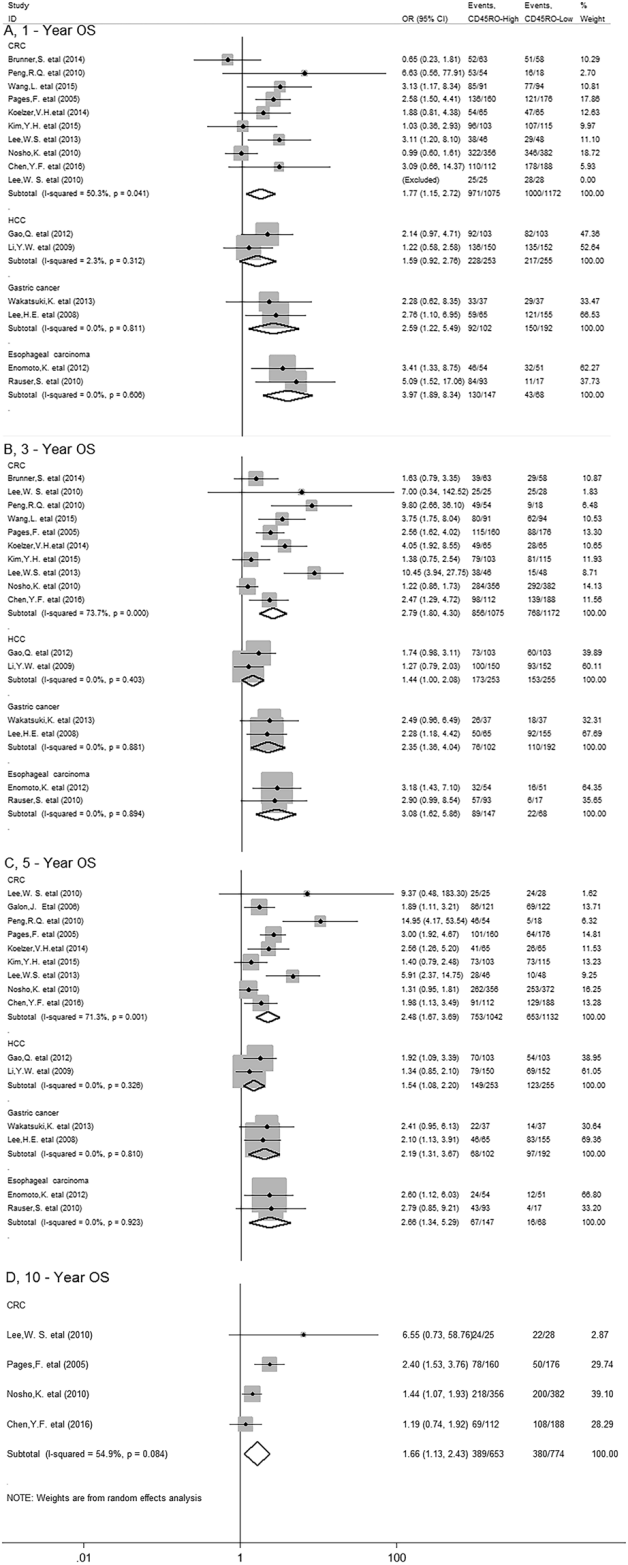
Stratified analyses describing ORs of the association between CD45RO^+^ T cell infiltration and OS at 1-year, 3-year, 5-year and 10-year.

In stratified analyses by cancer types, as shown in Fig. 3, pooled results showed that high density of CD45RO^+^ T cells significantly improved OS at 1-year (OR = 1.77, 95% CI 1.15 to 2.72, *P* = 0.009), 3-year (OR = 2.79, 95% CI 1.80 to 4.30, *P* = 0.000) 5-year (OR = 2.48, 95% CI 1.67 to 3.69, *P* = 0.000) and 10-year (OR = 1.66, 95% CI 1.13 to 2.43, *P* = 0.010) in colorectal cancer (CRC) as well as 1-year (OR = 2.59, 95% CI 1.22 to 5.49, *P* = 0.013), 3-year (OR = 2.35, 95% CI 1.38 to 4.04, *P* = 0.002) and 5-year OS (OR = 2.19, 95% CI 1.31 to 3.67, *P* = 0.003) in gastric cancer (GC); Similar results were observed between CD45RO^+^ T cells and 1-year (OR = 3.97, 95% CI 1.89 to 8.34, *P* = 0.000), 3-year (OR = 3.08, 95% CI 1.62 to 5.86, *P* = 0.001) and 5-year OS (OR = 2.66, 95% CI 1.34 to 5.29, *P* = 0.005) of esophageal carcinoma (EC) patients. However, in hepatocellular carcinoma (HCC), we found CD45RO^+^ T cell infiltration was significantly associated with improved 5-year (OR = 1.54, 95% CI 1.08 to 2.20, *P* = 0.016), but not with 1-year (OR = 1.59, 95% CI 0.92 to 2.76, *P* = 0.095) or 3-year OS (OR = 1.44, 95% CI 1.00 to 2.08, *P* = 0.050). By the way, there was only one study reporting the relevant data for OS in breast, ovarian, cervical, endometrial cancer and non-small cell lung cancer (NSCLC), gallbladder, renal cell carcinoma, diffuse large B-cell lymphomas, malignant pleural mesothelioma respectively, thus, we couldn’t get a combined result for them.

#### Disease-free survival (DFS)

**Figure 4:**
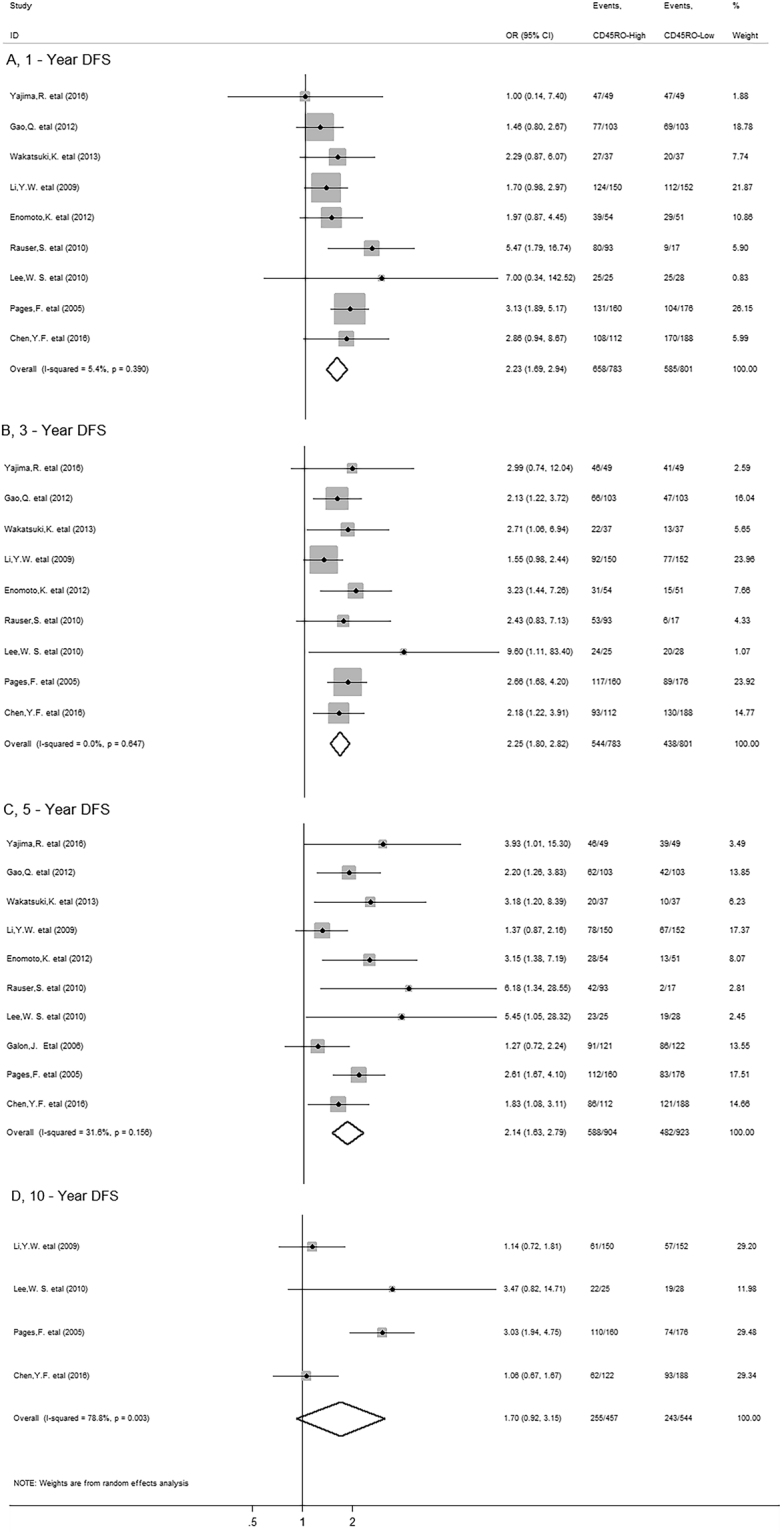
Forest plots describing ORs of the association between CD45RO^+^ T cell infiltration and DFS at 1-year, 3-year, 5-year and 10-year.

Meta-analysis showed that CD45RO^+^ T cell infiltration was significantly associated with improved 1-year (OR = 2.23, 95% CI 1.69 to 2.94, *P* = 0.000), 3-year (OR = 2.25, 95% CI 1.80 to 2.82, *P* = 0.000) and 5-year DFS (OR = 2.14, 95% CI 1.63 to 2.79, *P* = 0.000), but not with 10-year (OR = 1.70, 95% CI 0.92 to 3.15, *P* = 0.091) DFS in solid tumors (Fig. 4).

**Figure 5:**
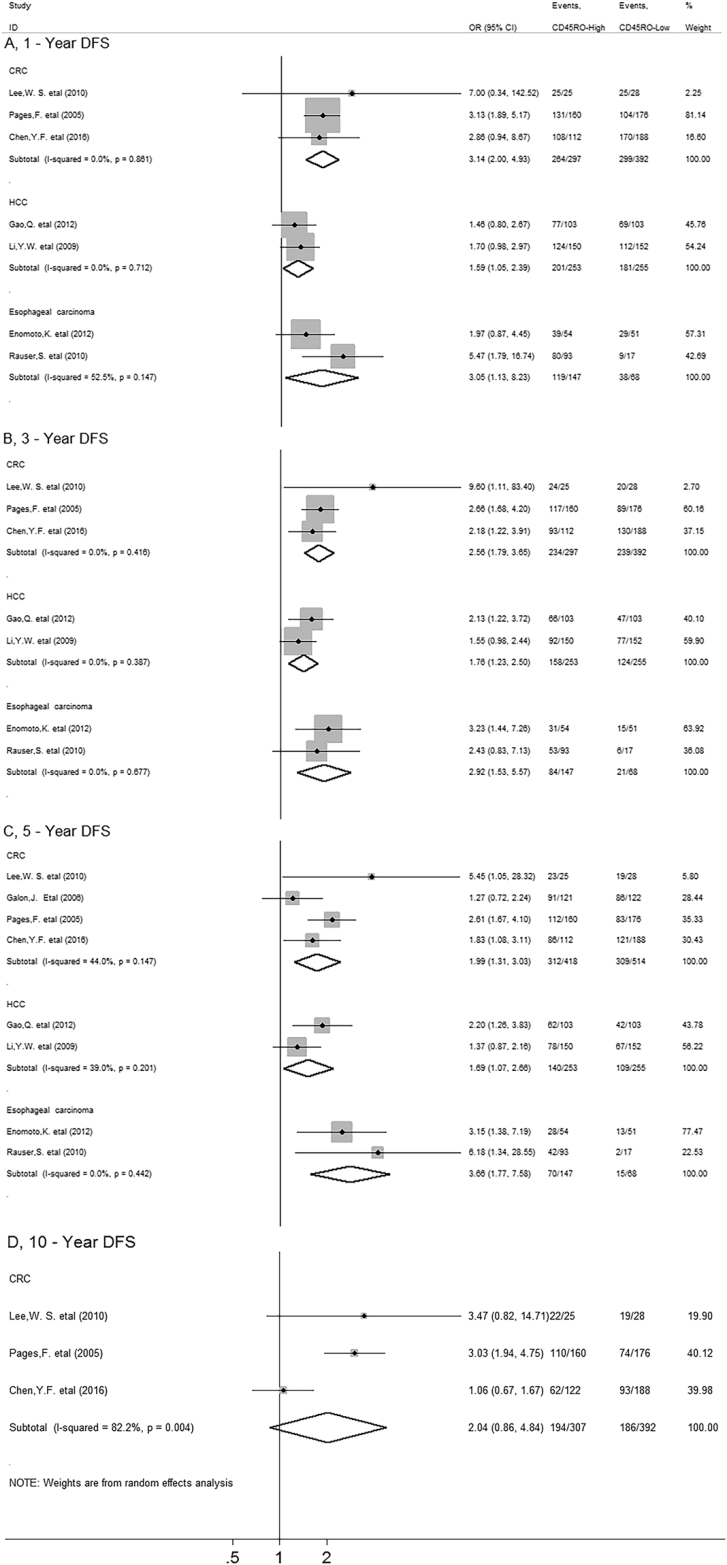
Stratified analyses describing ORs of the association between CD45RO^+^ T cell infiltration and DFS at 1-year, 3-year, 5-year and 10-year.

In stratified analyses by cancer types, as shown in Fig. 5, increased density of CD45RO^+^ T cells within tumor was significantly associated with better 1-year (OR = 3.14, 95% CI 2.00 to 4.93, *P* = 0.000), 3-year (OR = 2.56, 95% CI 1.79 to 3.65, *P* = 0.000) and 5-year DFS (OR = 1.99, 95% CI 1.31 to 3.03, *P* = 0.001), but not with 10-year DFS (OR = 2.04, 95% CI 0.86 to 4.84, *P* = 0.104) in colorectal cancer. CD45RO^+^ T cell infiltration also improved 1-year (OR = 1.59, 95% CI 1.05 to 2.39, *P* = 0.027), 3-year (OR = 1.76, 95% CI 1.23 to 2.50, *P* = 0.002) and 5-year DFS (OR = 1.69, 95% CI 1.07 to 2.66, *P* = 0.024) in HCC as well as 1-year (OR = 3.05, 95% CI 1.13 to 8.23, *P* = 0.028), 3-year (OR = 2.92, 95% CI 1.53 to 5.57, *P* = 0.001) and 5-year DFS (OR = 3.66, 95% CI 1.77 to 7.58, *P* = 0.000) in esophageal carcinoma.

**Figure 6:**
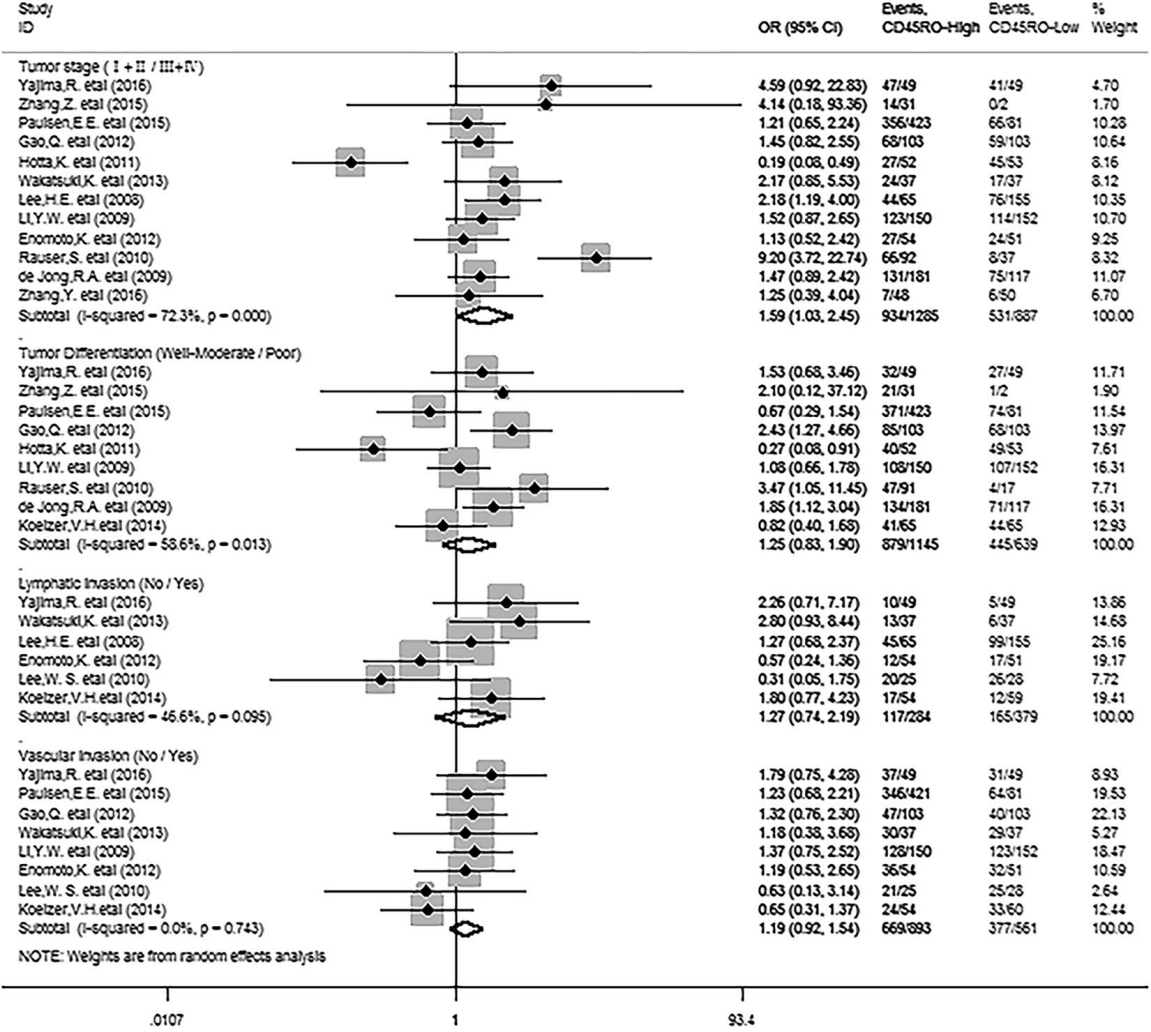
Forest plots indicating ORs of the association between CD45RO^+^ T cell infiltration and clinicopathological features.

We next investigated whether CD45RO^+^ T cell infiltration was associated with clinicopathological features such as TNM stage, tumor differentiation, Lymphatic invasion and vascular invasion of solid tumor. We found that CD45RO^+^ T cell infiltration was significantly inversely correlated with TNM stage (OR = 1.59, 95% CI 1.03 to 2.45, *P* = 0.038), but not with tumor differentiation (OR = 1.25, 95% CI 0.83 to 1.90, *P* = 0.285), lymphatic invasion (OR = 1.27, 95% CI 0.74 to 2.19, *P* = 0.385), or vascular invasion (OR = 1.19, 95% CI 0.92 to 1.54, *P* = 0.191) of patients (Fig. 6).

#### Sensitivity analysis

Sensitivity analyses were used to determine the influence of individual studies on the overall OR. As a result, the plot showed that all the individual studies had no important impact on the result for OS or DFS (data not shown).

#### Publication bias

Funnel plot and Egger’s test were performed to assess the publication bias of this meta-analysis. No significant publication bias existed between CD45RO^+^ T cell infiltration and OS or DFS in cancer patients (data not shown).

## Discussion

As memory cells may prevent recurrence in cancer patients, CD45RO expression in TILs might predict immune response to recurrence after tumor resection. Although many studies have associated tumor-infiltrating CD45RO^+^ T cells and prognosis of solid tumors, their results were not consistent even controversial. In the present meta-analysis, we found that CD45RO^+^ T cell infiltration had a positive prognostic effect associated with survival in many types of solid tumors especially in CRC, HCC, GC and EC. In addition, increased density of CD45RO^+^ T cells was significantly inversely associated with TNM stage of solid tumor. We believe our study provides meaningful statistical evidence to report the important prognostic value of CD45RO^+^ T cell infiltration as a cancer fighter in patients with solid tumor for the first time.

However, the exact mechanisms underlying CD45RO^+^ T cell – mediated survival improvement still remain unclear. The possible explanations are as follows: it may partially relate to the features of CD45RO^+^ T cells, as they: (1) are the hallmark of adaptive immunity; (2) display a low-activation threshold; (3) vigorously proliferate despite minimal co-stimulation; and (4) persist over a life-time with stem cell-like multipotency and self-renewal characteristics^35^. More importantly, tumor-infiltrating CD45RO^+^ T cells which experienced tumor antigens are probably effector memory CD8^+^ T cells (CD8^+^ Tem), can secret amount of INF-γ and granzyme to induce potent anti-tumor immune responses. *In situ* immune reactions can reflect and influence systematic anti-tumor capability. After the resection of primary tumor, central memory T cells (Tcm), as another subset of CD45RO^+^ memory T cells, increase and home to the secondary lymphatic organ and exhibit persistent anti-tumor effect via various mechanisms including INF-γ production. Thus, it is reasonable to speculate that the CD45RO^+^ T cells are able to respond to and eliminate residue tumor cells therefore improving survival.

Some limitations should be noted from this meta-analysis. First, significant heterogeneity observed across studies cannot be completely accounted despite the use of appropriate meta-analytic techniques with random-effect models. Second, there was only one study reporting the relevant data for OS in several cancers, thus, we couldn’t get a combined result for them. Finally, studies with negative results or small sample size may not be published, which can cause potential publication bias.

In conclusion, CD45RO^+^ memory T lymphocyte infiltration is associated with favorable clinical outcome of patients with solid tumor, implicating that these cells might be a potential biomarker for prognostic prediction for human solid malignances.

## Electronic supplementary material

Supplementary Information

## References

[1] Motz GT, Coukos G (2011). The parallel lives of angiogenesis and immunosuppression: cancer and other tales. Nat Rev Immunol.

[2] Hanahan D, Weinberg RA (2011). Hallmarks of cancer: the next generation. Cell.

[3] Leffers N (2009). Prognostic significance of tumor-infiltrating T-lymphocytes in primary and metastatic lesions of advanced stage ovarian cancer. Cancer immunology, immunotherapy: CII.

[4] Michie CA, McLean A, Alcock C, Beverley PC (1992). Lifespan of human lymphocyte subsets defined by CD45 isoforms. Nature.

[5] Stang A (2010). Critical evaluation of the Newcastle-Ottawa scale for the assessment of the quality of nonrandomized studies in meta-analyses. European journal of epidemiology.

[6] Higgins JP, Thompson SG, Deeks JJ, Altman DG (2003). Measuring inconsistency in meta-analyses. Bmj.

[7] Kuritz SJ, Landis JR, Koch GG (1988). A general overview of Mantel-Haenszel methods: applications and recent developments. Annual review of public health.

[8] DerSimonian R, Kacker R (2007). Random-effects model for meta-analysis of clinical trials: an update. Contemporary clinical trials.

[9] Egger M, Davey Smith G, Schneider M (1997). & Minder, C. Bias in meta-analysis detected by a simple, graphical test. Bmj.

[10] Yajima R (2016). Tumor-infiltrating CD45RO(+) memory cells are associated with a favorable prognosis breast cancer. Breast cancer.

[11] Zhang Z (2015). Infiltration of dendritic cells and T lymphocytes predicts favorable outcome in epithelial ovarian cancer. Cancer gene therapy.

[12] Paulsen EE (2015). CD45RO(+) Memory T Lymphocytes–a Candidate Marker for TNM-Immunoscore in Squamous Non-Small Cell Lung Cancer. Neoplasia.

[13] Gao Q (2012). Infiltrating memory/senescent T cell ratio predicts extrahepatic metastasis of hepatocellular carcinoma. Ann Surg Oncol.

[14] Hotta K (2011). Prognostic significance of CD45RO+ memory T cells in renal cell carcinoma. British journal of cancer.

[15] Wakatsuki K (2013). Clinical impact of tumor-infiltrating CD45RO(+) memory T cells on human gastric cancer. Oncology reports.

[16] Lee HE (2008). Prognostic implications of type and density of tumour-infiltrating lymphocytes in gastric cancer. British journal of cancer.

[17] Li YW (2009). Tumor-infiltrating macrophages can predict favorable prognosis in hepatocellular carcinoma after resection. Journal of cancer research and clinical oncology.

[18] Enomoto K (2012). Prognostic importance of tumour-infiltrating memory T cells in oesophageal squamous cell carcinoma. Clin Exp Immunol.

[19] Chang KC, Huang GC, Jones D, Lin YH (2007). Distribution patterns of dendritic cells and T cells in diffuse large B-cell lymphomas correlate with prognoses. Clin Cancer Res.

[20] Anraku M (2008). Impact of tumor-infiltrating T cells on survival in patients with malignant pleural mesothelioma. The Journal of thoracic and cardiovascular surgery.

[21] Rauser S (2010). High number of CD45RO+ tumor infiltrating lymphocytes is an independent prognostic factor in non-metastasized (stage I-IIA) esophageal adenocarcinoma. BMC Cancer.

[22] Brunner SM (2014). Prognosis according to histochemical analysis of liver metastases removed at liver resection. The British journal of surgery.

[23] de Jong RA (2009). Presence of tumor-infiltrating lymphocytes is an independent prognostic factor in type I and II endometrial cancer. Gynecologic oncology.

[24] Zhang, Y. *et al*. Prognostic significance of immune cells in the tumor microenvironment and peripheral blood of gallbladder carcinoma patients. *Clinical & translational oncology: official publication of the Federation of Spanish Oncology Societies and of the National Cancer Institute of Mexico*, doi:10.1007/s12094-016-1553-6 (2016). 10.1007/s12094-016-1553-6 27718154

[25] Lee WS, Park S, Lee WY, Yun SH, Chun HK (2010). Clinical impact of tumor-infiltrating lymphocytes for survival in stage II colon cancer. Cancer.

[26] Galon J (2006). Type, density, and location of immune cells within human colorectal tumors predict clinical outcome. Science.

[27] Peng RQ (2010). Co-expression of nuclear and cytoplasmic HMGB1 is inversely associated with infiltration of CD45RO+ T cells and prognosis in patients with stage IIIB colon cancer. BMC Cancer.

[28] Wang L, Zhai ZW, Ji DB, Li ZW, Gu J (2015). Prognostic value of CD45RO(+) tumor-infiltrating lymphocytes for locally advanced rectal cancer following 30 Gy/10f neoadjuvant radiotherapy. International journal of colorectal disease.

[29] Pages F (2005). Effector memory T cells, early metastasis, and survival in colorectal cancer. The New England journal of medicine.

[30] Koelzer VH (2014). CD8/CD45RO T-cell infiltration in endoscopic biopsies of colorectal cancer predicts nodal metastasis and survival. Journal of translational medicine.

[31] Kim Y, Bae JM, Li G, Cho NY, Kang GH (2015). Image analyzer-based assessment of tumor-infiltrating T cell subsets and their prognostic values in colorectal carcinomas. PLoS One.

[32] Lee WS, Kang M, Baek JH, Lee JI, Ha SY (2013). Clinical impact of tumor-infiltrating lymphocytes for survival in curatively resected stage IV colon cancer with isolated liver or lung metastasis. Ann Surg Oncol.

[33] Nosho K (2010). Tumour-infiltrating T-cell subsets, molecular changes in colorectal cancer, and prognosis: cohort study and literature review. J Pathol.

[34] Chen Y (2016). A Novel Immune Marker Model Predicts Oncological Outcomes of Patients with Colorectal Cancer. Ann Surg Oncol.

[35] Woodland DL, Kohlmeier JE (2009). Migration, maintenance and recall of memory T cells in peripheral tissues. Nat Rev Immunol.

