# Supplementary material for: Tumor-infiltrating CD45RO+ Memory T Lymphocytes Predict Favorable Clinical Outcome in Solid Tumors
Source: Sci Rep. 2017 Sep 4;7:10376. doi: 10.1038/s41598-017-11122-2 (PMC5583330; doi:10.1038/s41598-017-11122-2)
Supplement: Supplementary file 1 — Supplementary Information [file 41598_2017_11122_MOESM1_ESM.docx]

**Tumor-infiltrating CD45RO^+^ Memory T Lymphocytes Predict Favorable Clinical Outcome in Solid Tumors**

Guoming Hu ^1,^*, Shimin Wang ^2^

^1^ Department of General Surgery (Breast and Thyroid Surgery), Shaoxing People’s Hospital; Shaoxing Hospital of Zhejiang University; 312000, Zhejiang, China.

^2^ Department of Nephrology, Shaoxing People’s Hospital; Shaoxing Hospital of Zhejiang University; 312000, Zhejiang, China.

*Correspondence: E-mail: hgmplj@126.com

**Table S1.** Characteristics of the included studies for OR analysis of clinicopathological features.

| **Study** | **Year** | **Tumor type** | **No. of Patients** | **CD45RO^+^ T: High/Low** | **Tumor stage** | **Ⅰ+Ⅱ /**  **Ⅲ+Ⅳ** | **Lymphatic invasion (No / Yes)** | **Vascular invasion (No / Yes)** | **Tumor Differentiation**  **(Well-moderate/poor)** |
| --- | --- | --- | --- | --- | --- | --- | --- | --- | --- |
| Yajima,R. etal ^10^ | 2016 | Breast cancer | 98 | 49/49 | Ⅰ - Ⅲ | H: (47/2) L: (41/8) | H: (39/10); L: (44/5) | H: (12/37);  L: (18/31) | H: (32/17)  L: (27/22) |
| Zhang,Z. etal ^11^ | 2015 | Ovarian cancer | 33 | 31/2 | Ⅰ - Ⅳ | H: (14/17) L: (0/2) | NR | NR | H: (21/10) L: (1/1) |
| Paulsen,E.E. etal ^12^ | 2015 | Non-small Cell Lung Cancer | 504 | 423/81 | Ⅰ - Ⅲ | H: (356/67) L: (66/15) | NR | H: (75/346);  L: (17/64) | H: (371/52)  L: (74/7) |
| Gao,Q. etal ^13^ | 2012 | Hepatocellular carcinoma | 206 | 103/103 | Ⅰ - Ⅲ | H: (68/35) L: (59/44) | NR | H: (56/47);  L: (63/40) | H: (85/18)  L: (68/35) |
| Hotta,K. etal ^14^ | 2011 | Renal cell carcinoma | 105 | 52/53 | Ⅰ - Ⅳ | H: (27/25) L: (45/8) | NR | NR | H: (40/12)  L: (49/4) |
| Wakatsuki,K. etal ^15^ | 2013 | Gastric cancer | 74 | 37/37 | Ⅰ- Ⅳ | H: (24/13) L: (17/20) | H: (24/13); L: (31/6) | H: (7/30);  L: (8/29) | NR |
| Lee,H.E. etal ^16^ | 2008 | Gastric cancer | 220 | 65/155 | Ⅰ- Ⅳ | H: (44/21) L: (76/79) | H: (20/45);  L: (56/99) | NR | NR |
| Li,Y.W. etal ^17^ | 2009 | Hepatocellular carcinoma | 302 | 150/152) | Ⅰ - Ⅲ | H: (123/27) L: (114/38) | NR | H: (22/128);  L: (29/123) | H: (108/42)  L: (107/45) |
| Enomoto,K. etal ^18^ | 2012 | Esophageal carcinoma | 105 | 54/51 | Ⅰ- Ⅳ | H: (27/27) L: (24/27) | H: (42/12);  L: (34/17) | H: (18/36);  L: (19/32) | NR |
| Rauser,S. etal ^21^ | 2010 | Esophageal carcinoma | 110 | 93/17 | Ⅰ- Ⅳ | H: (66/26) L: (8/29) | NR | NR | H: (47/44)  L: (4/13) |
| de Jong,R.A. etal ^23^ | 2009 | Endometrial cancer | 298 | 181 /117 | Ⅰ- Ⅳ | H: (131/50) L: (75/42) | NR | NR | H: (134/47)  L: (71/46) |
| Lee,W. S. etal ^25^ | 2010 | Colorectal cancer | 53 | 25/28 | Ⅱ | NR | H: (5/20);  L: (2/26) | H: (4/21);  L: (3/25) | NR |
| Zhang,Y. etal ^24^ | 2016 | Gallbladder carcinoma | 98 | 48/50 | Ⅰ- Ⅳ | H: (7/41) L: (6/44) | NR | NR | NR |
| Koelzer,V.H.etal ^30^ | 2014 | Colorectal cancer | 130 | 65/65 | Ⅰ- Ⅳ | NR | H: (37/17);  L: (47/12) | H: (30/24);  L: (27/33) | H: (41/24)  L: (44/21) |

T: primary tumor; N: lymph node; M: metastasis; H: high; L: low; NR: not reported.
